# Supplementary material for: Nutritional and host environments determine community ecology and keystone species in a synthetic gut bacterial community
Source: Nat Commun. 2023 Aug 8;14:4780. doi: 10.1038/s41467-023-40372-0 (PMC10409746; doi:10.1038/s41467-023-40372-0)
Supplement: Supplementary file 1 — Supplementary Information [file 41467_2023_40372_MOESM1_ESM.pdf]

Supplemental Information

Supplemental Figures

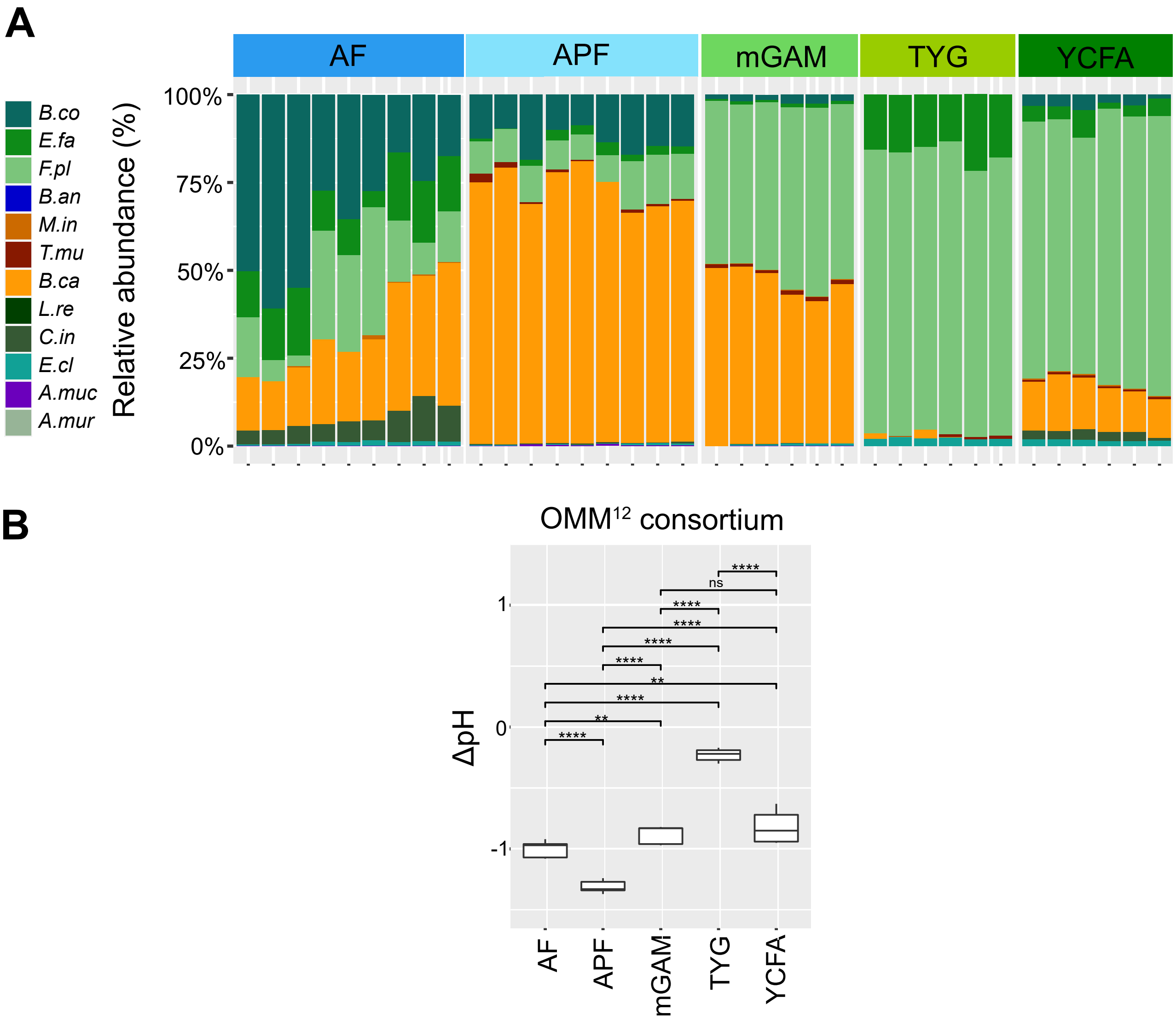

Fig. S1. OMM^{12} community composition and ΔpH values in five different culture media. Relative abundance of strains (**A**) after four days of serial dilution in batch culture was determined by qPCR as normalized 16S rRNA copies per ml culture. Adaptations to the previously published batch culture procotol (Weiss et al., ISME J, 2022) include the inoculation from frozen inocula stocks and a shortened passaging period of four instead of ten days. Using a two-sided t-test, ΔpH values (pH of fresh medium subtracted from pH of the community spent culture supernatant on day four) were compared between the communities grown in the different media (N=9 for AF and APF medium, N=6 for mGAM, TYG and YCFA medium), p values are denoted as ns = not significant, * < 0.05, ** < 0.01, *** < 0.005, **** < 0.001. Median ΔpH values (black line) are given with the corresponding upper and lower percentile (box, whiskers indicate 1.5 times interquartile range) (**B**).

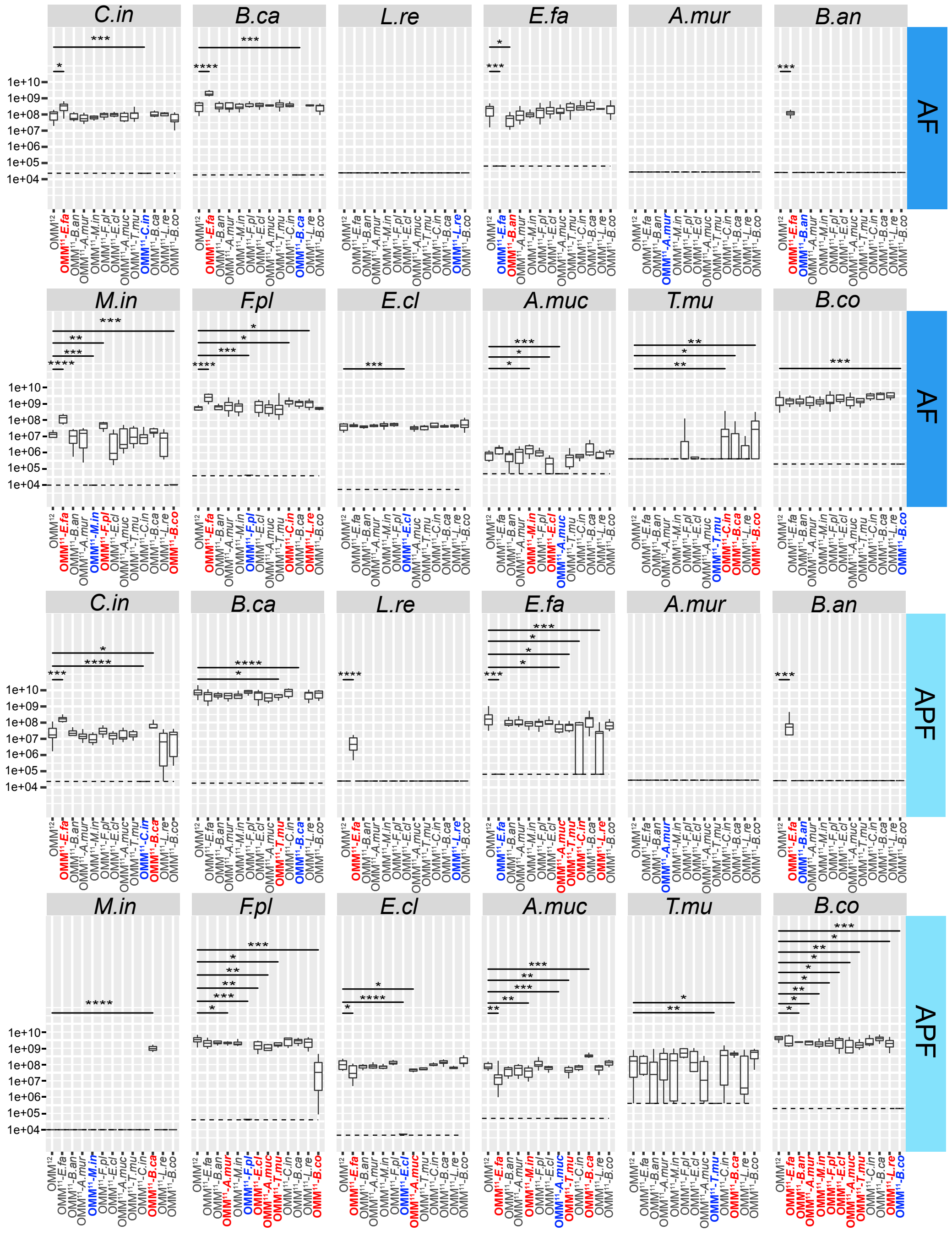

Fig. S2. Community composition of all dropout communities and the full community in AF and APF medium. Absolute abundance of strains after four days of serial dilution in batch culture was determined by qPCR as normalized 16S rRNA copies per ml culture. Median absolute abundances (black line) are shown with the corresponding upper and lower percentile (box, whiskers indicate 1.5 times interquartile range). The strain specific detection limit is shown as dotted line. Using a two-sided Wilcoxon test absolute abdundances were compared between the communities (N=9 each), p values are denoted as * < 0.05, ** < 0.01, *** < 0.005, **** < 0.001. Non-significantly changed comparisons are not shown.

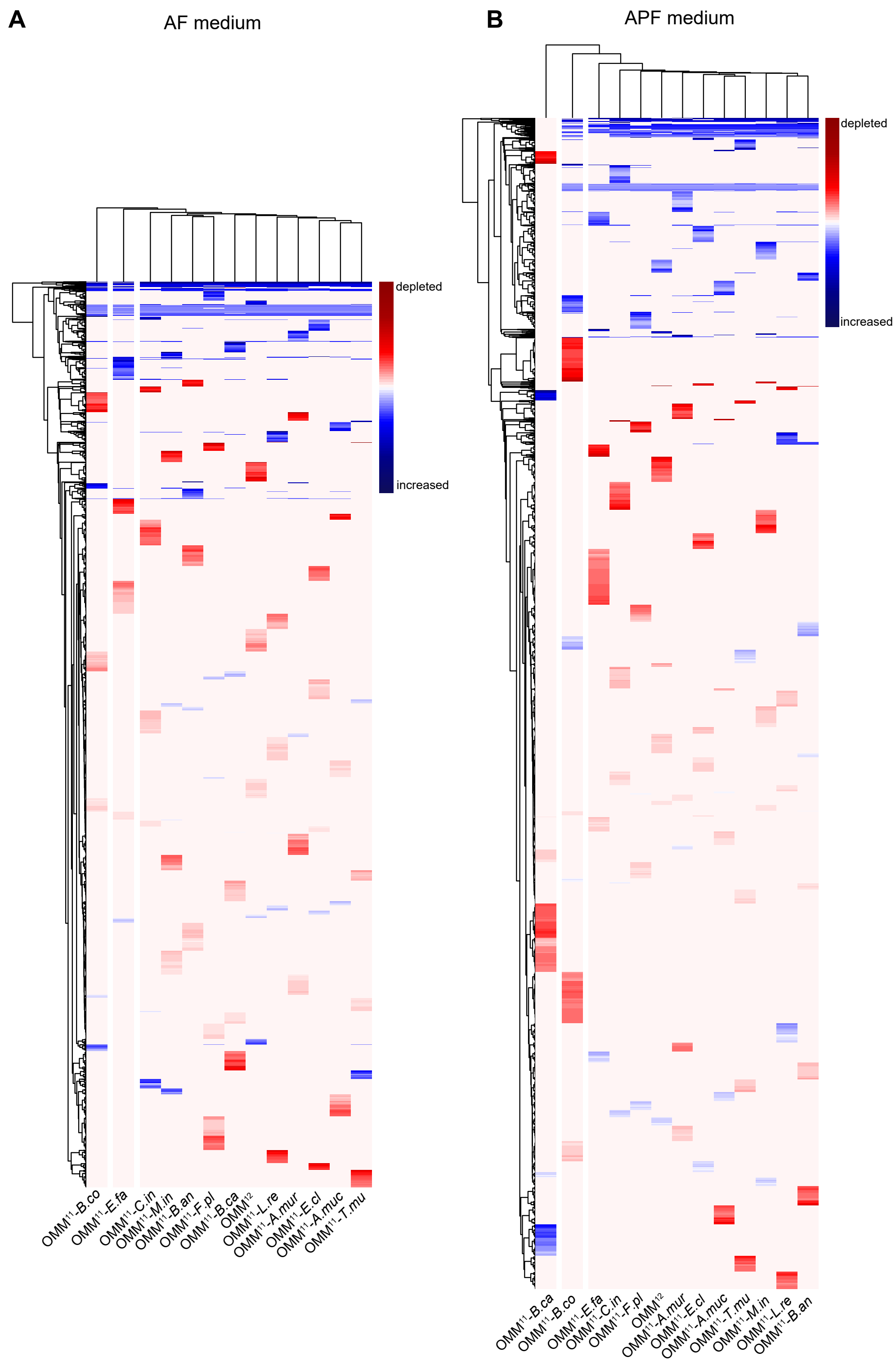

Fig. S3. Untargeted metabolomics analysis of community spent media. Metabolomic profiles after community growth to stationary phase in AF medium (**A**) and APF medium (**B**) were determined by untargeted MS. All metabolomic features that significantly changed in comparison to the medium blank for at least one of the 13 communities are shown (rows, source data table 2). Levels decreased and levels increased compared to fresh AF or APF medium as determined by the relative foldchange are shown in red and blue, respectively. Hierarchical clustering of community specific profiles revealed more pronounced differences in profiles for communities lacking *B. coccoides* and *E. faecalis* in AF medium and the *B. caecimuris* dropout community in APF medium.

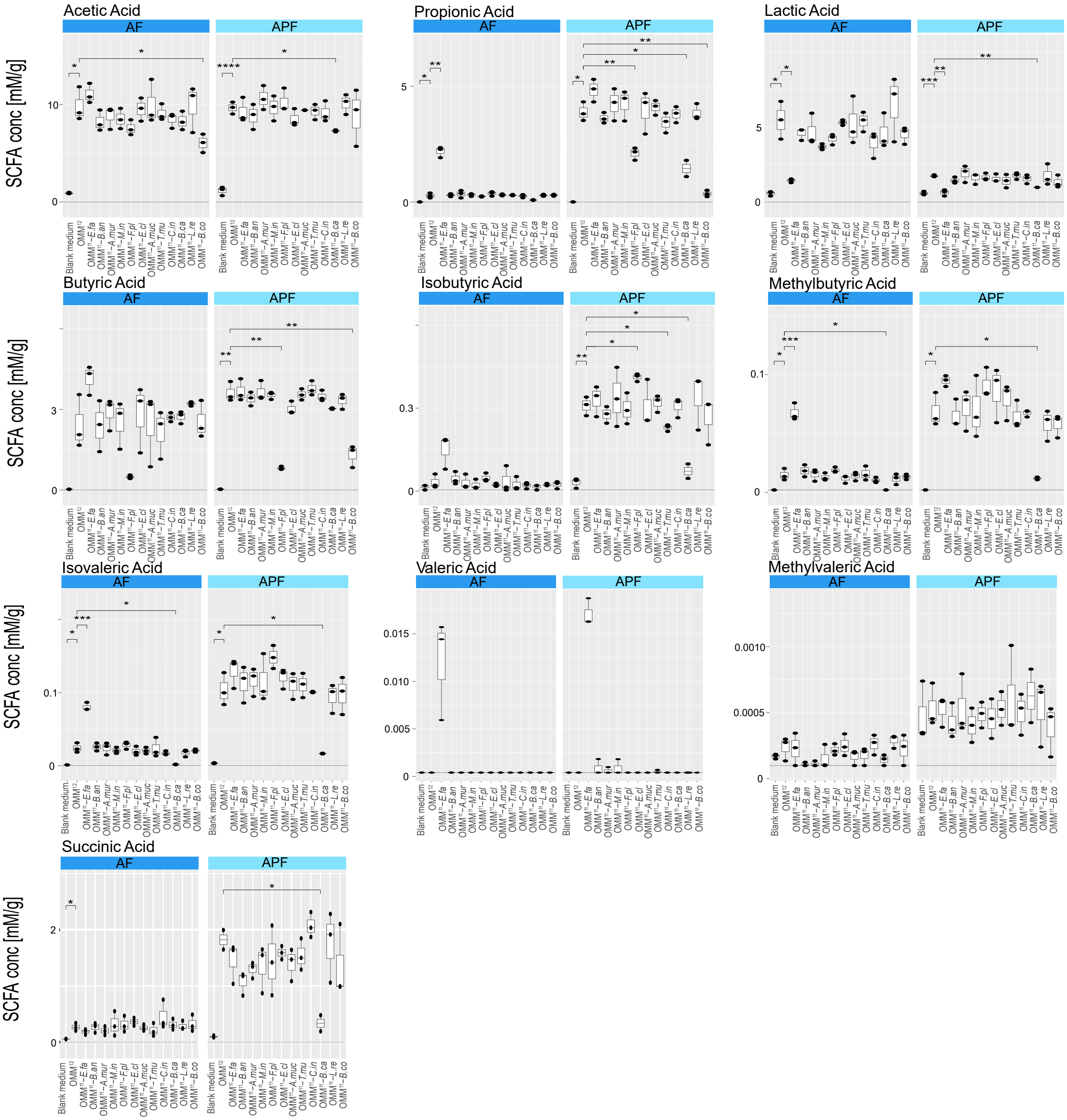

Fig. S4. SCFA concentrations in community spent media. SCFA concentrations were determined by targeted metabolomics analysis of community spent media and fresh media (AF, APF, N=3 each) and are shown as median (black line) with the corresponding upper and lower percentile (box, whiskers indicate 1.5 times interquartile range, points outside this range are considered outliers). Using a two-sided t-test the SCFA concentrations in fresh media as well as in all dropout communities were compared to the corresponding concentration in the full consortium. p values are denoted as ns = not significant, * < 0.05, ** < 0.01, *** < 0.005, **** < 0.001. Non-significantly changed comparisons are not shown.

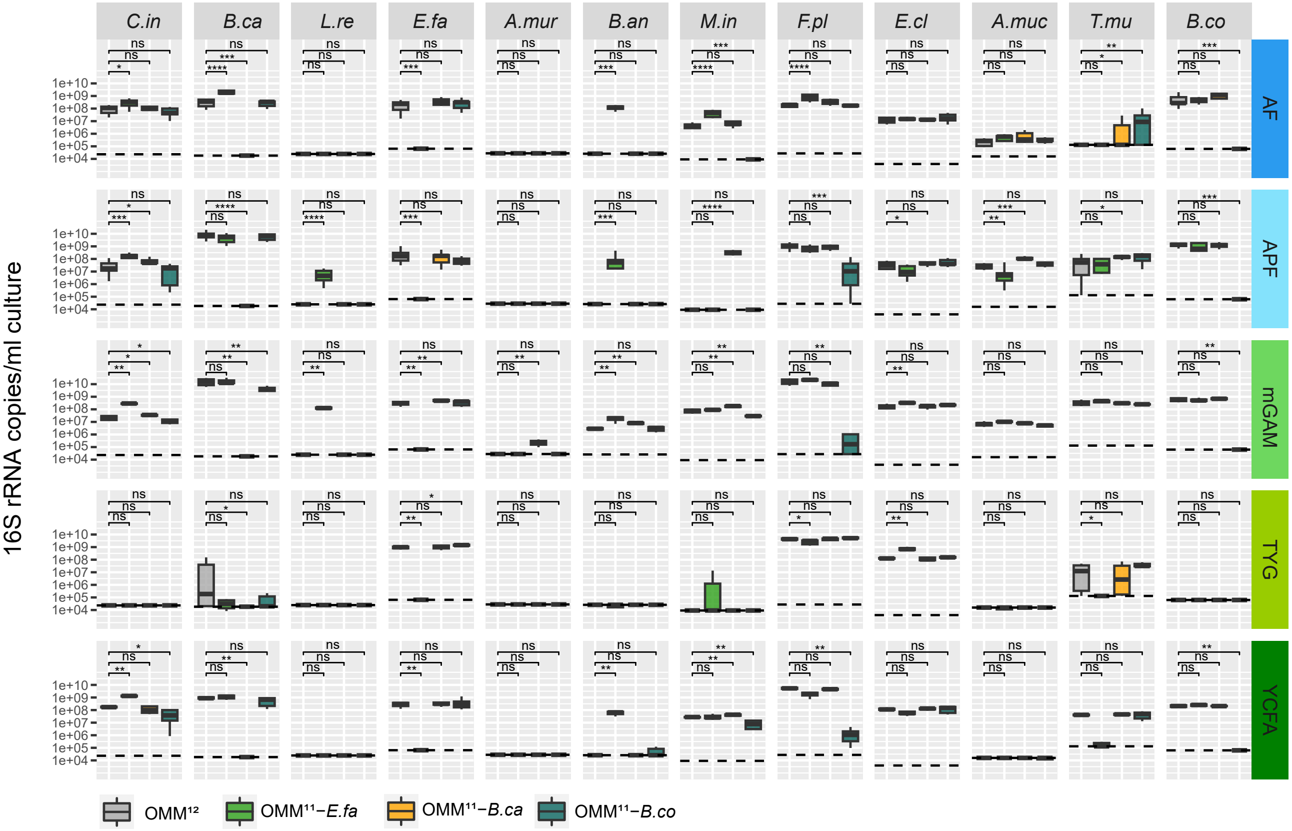

Fig. S5. Community assembly of consortia lacking three context- dependent keystone species in different culture media. Absolute abundance of strains after four days of serial dilution in batch culture in AF, APF, mGAM, TYG and YCFA medium (N=9 for AF and APF medium, N=6 for mGAM, TYG and YCFA medium) was determined by qPCR as normalized 16S rRNA copies per ml culture for the full consortium and communities lacking the three identified context-dependent keystone species *E. faecalis*, *B. caecimuris*, *B. coccoides*. Median absolute abundances (black line) are shown with the corresponding upper and lower percentile (box, whiskers indicate 1.5 times interquartile range). The strain specific detection limit is shown as dotted line. Using a two-sided Wilcoxon test, absolute abundances of the individual strains were compared between the full consortium and the corresponding dropout communities, p values are denoted as ns = not significant, $* < 0.05$, $** < 0.01$, $***< 0.005$, $**** < 0.001$

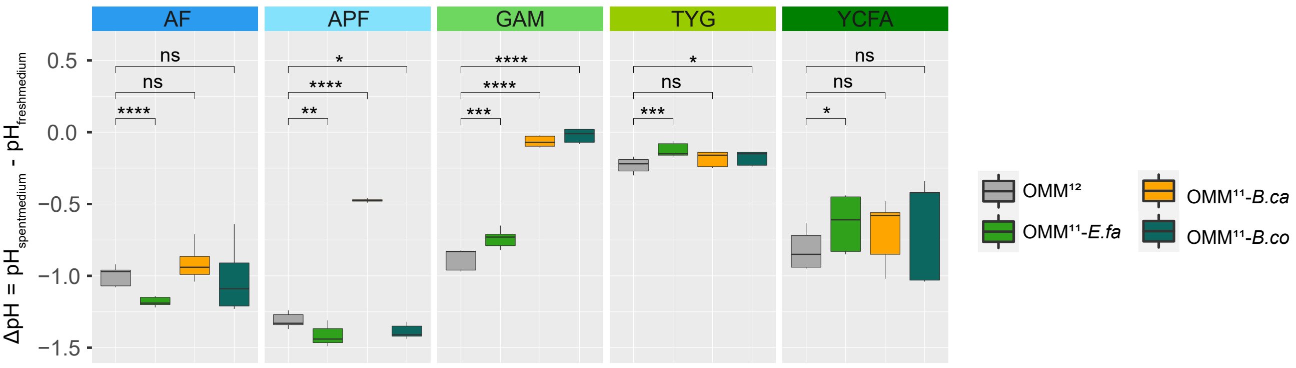

Fig. S6. ΔpH values of consortia lacking three context- dependent keystone species in different culture media. ΔpH values (pH of fresh medium subtracted from pH of the community spent culture supernatant on day four) were compared between the dropout and the full communities grown in AF, APF, mGAM, TYG and YCFA medium (N=9 for AF and APF medium, N=6 for mGAM, TYG and YCFA medium). Median ΔpH values (black line) are given with the corresponding upper and lower percentile (box, whiskers indicate 1.5 times interquartile range). Using a two-sided t-test the delta-pH values in the dropout communities were compared to the full consortium. p values are denoted as ns = not significant, * < 0.05, ** < 0.01, ***< 0.005, **** < 0.001

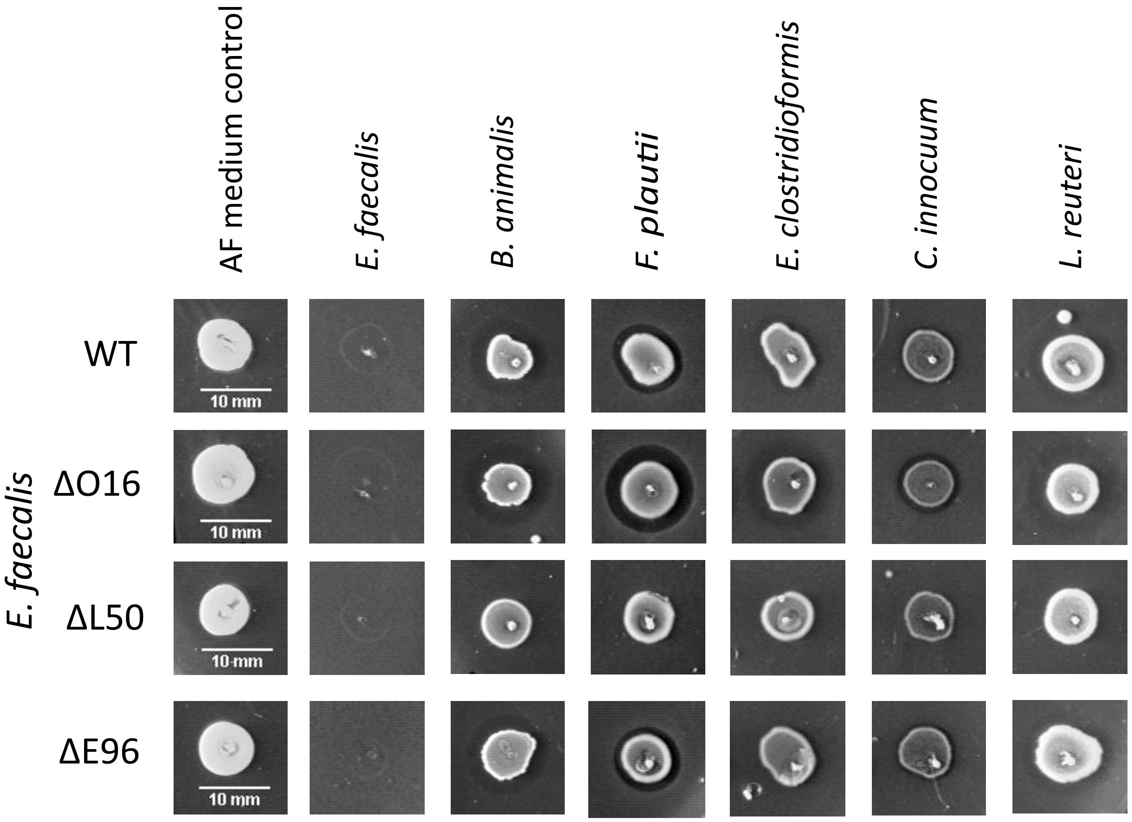

Fig. S7. Phenotyping approach of *E. faecalis* wildtype and mutant strains. Spot assays were used to test for the production of antibacterial compounds. *E. faecalis* wildtype and mutant strains lacking the loci for production of enterocin O16, enterocin L50 (A and B) and enterocin E96 were spotted onto a bacterial lawn of the initially susceptible strains (Weiss et al., ISME J, 2022): *B. animalis*, *F. plautii*, *E. clostridioformis*, *C. innocuum* and *L. reuteri*; as well as on a lawn of *E. faecalis* wildtype and AF medium as control. The *E. faecalis* ΔL50 mutant strain was the only strain that did not show clear inhibition zones on any of the tested other strains, identifying enterocin L50 A and B as the inhibiting enterocin.

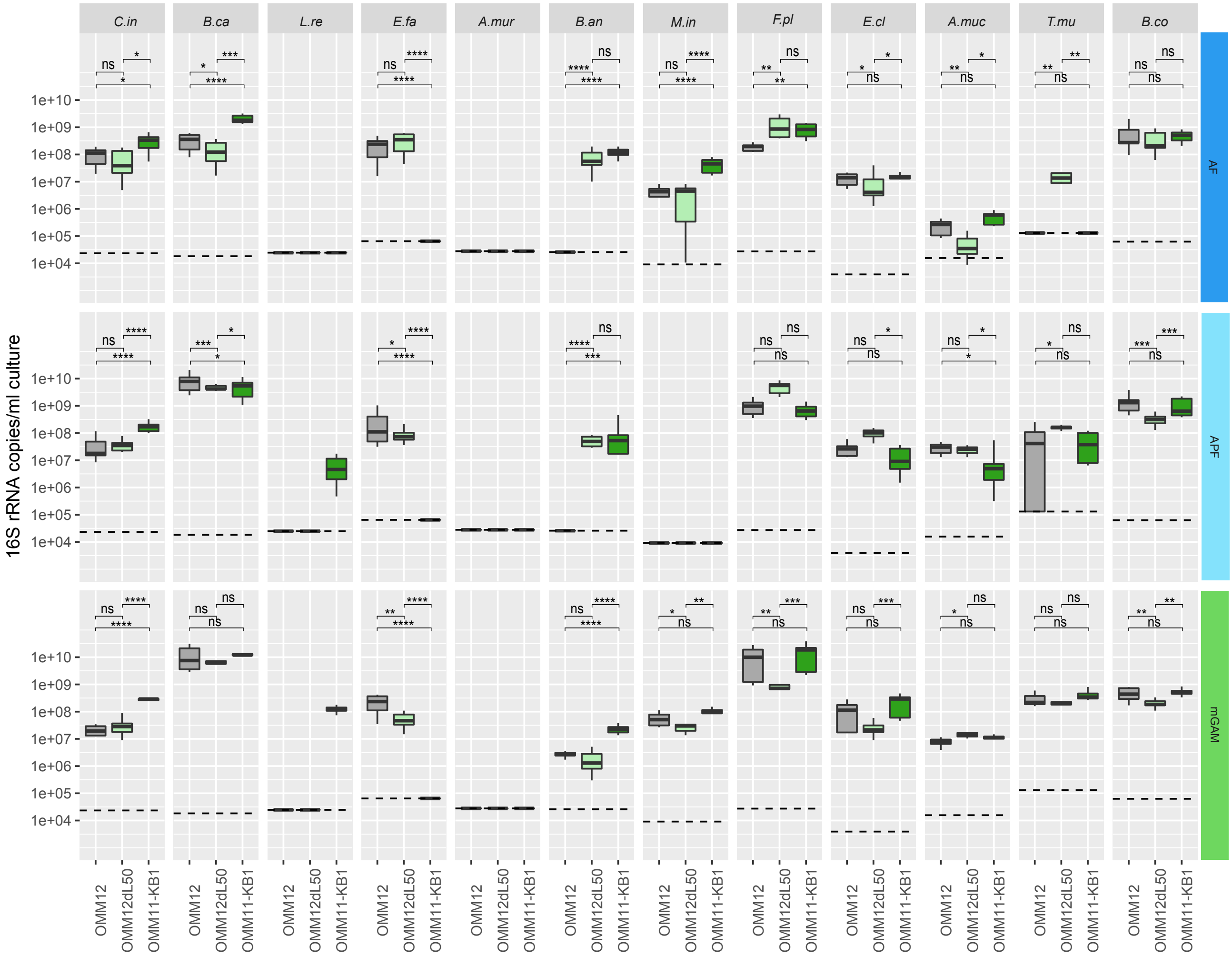

Fig. S8. Influence of enterocin L50 on community assembly. Absolute abundance of strains after four days of serial dilution in batch culture in AF, APF and mGAM medium was determined by qPCR as normalized 16S rRNA copies per ml culture for the full consortium, a community including the *E. faecalis* ΔL50 mutant strain and a *E. faecalis* dropout community. Median absolute abundances (black line) are shown with the corresponding upper and lower percentile (box, whiskers indicate 1.5 times interquartile range) for all individual strains. The strain specific detection limit is shown as dotted line. Using a two-sided Wilcoxon test, absolute abundances of the individual strains were compared between the full consortium and the *E. faecalis* ΔL50 mutant strain community, or the dropout community (N=9 each), p values are denoted as ns = not significant, * $<$ 0.05, ** $<$ 0.01, *** $<$ 0.005.

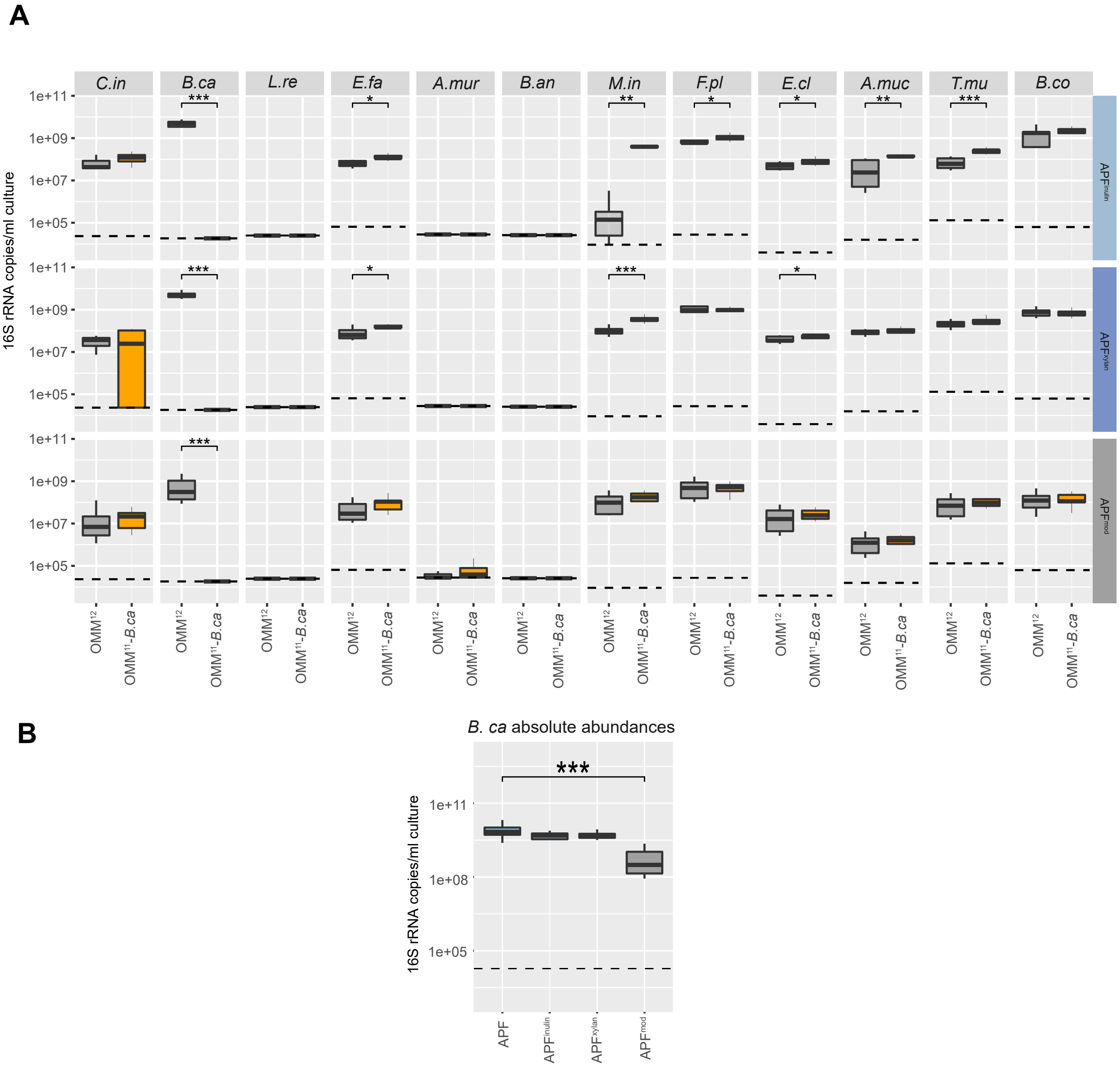

Fig. S9. Influence of polysaccharides in APF medium on community assembly and *B. caecimuris* abundance. Absolute abundance of strains after four days of serial dilution in batch culture in APF^{inulin}, APF^{xylan} and APF^{mod} (without polysaccharides inulin and xylan) was determined by qPCR as normalized 16S rRNA copies per ml culture **(A)**. Absolute abundances of *B. caecimuris* in APF, APF^{inulin}, APF^{xylan} and APF^{mod} in the full community are summarized overview **(B)**. Median absolute abundances (black line) are shown with the corresponding upper and lower percentile (box, whiskers indicate 1.5 times interquartile range) for all individual strains. The strain specific detection limit is shown as dotted line. Using a two-sided Wilcoxon test, absolute abundances of the individual strains were compared between the full consortium and the corresponding dropout communities (N=9 each), p values are denoted as ns = not significant, * $<$ 0.05, ** $<$ 0.01, *** $<$ 0.005. Non-significantly changed comparisons are not shown.

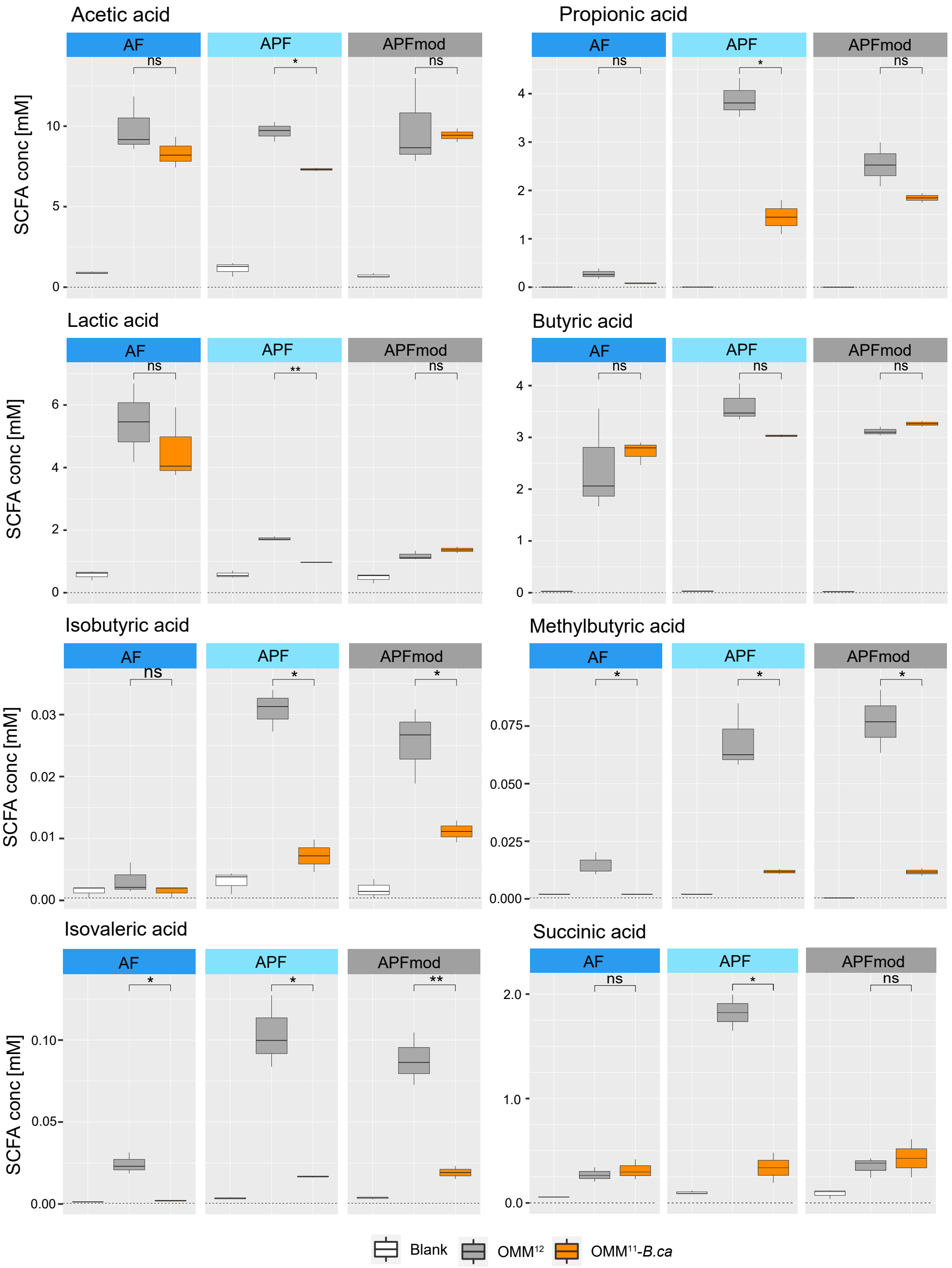

Fig. S10. SCFA concentration in *B. caecimuris* dropout communities in AF, APF and APF^{mod}. SCFA concentrations were determined by targeted metabolomics analysis of community spent media and fresh media (N=3 each) and are shown as median (black line) with the corresponding upper and lower percentile (box, whiskers indicate 1.5 times interquartile range). Using a two-sided t-test the SCFA concentrations in the *B. caecimuris* dropout communities grown in AF, APF or APF^{mod} medium were compared to the SCFA concentration in spent media of the full communities. p values are denoted as ns = not significant, * < 0.05, ** < 0.01.

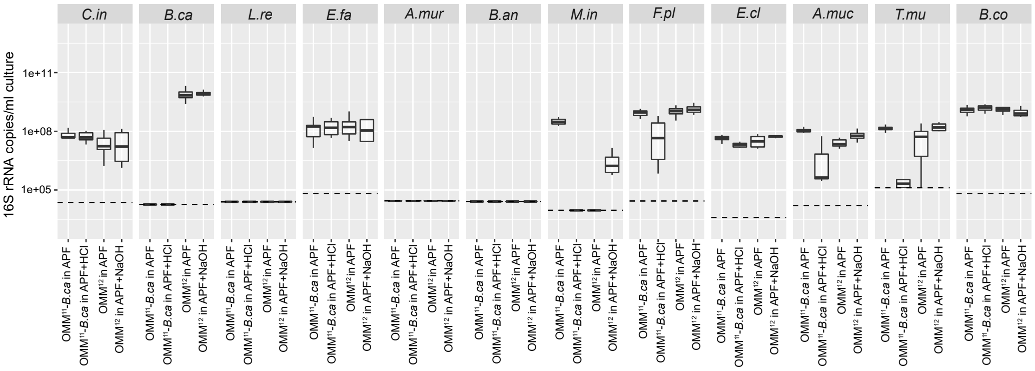

Fig. S11. Absolute abundance of community members of the full consortium vs. the *B. caecimuris* dropout community grown in pH-modified APF. The pH of the APF medium was modified with NaOH and HCl eight hours after each passage. Absolute abundance of the individual strains was determined by qPCR as normalized 16S rRNA copies per ml culture on day four of cultivation (N=9 each). Median absolute abundances (black line) are shown with the corresponding upper and lower percentile (box, whiskers indicate 1.5 times interquartile range). The strain specific detection limit is shown as dotted line.

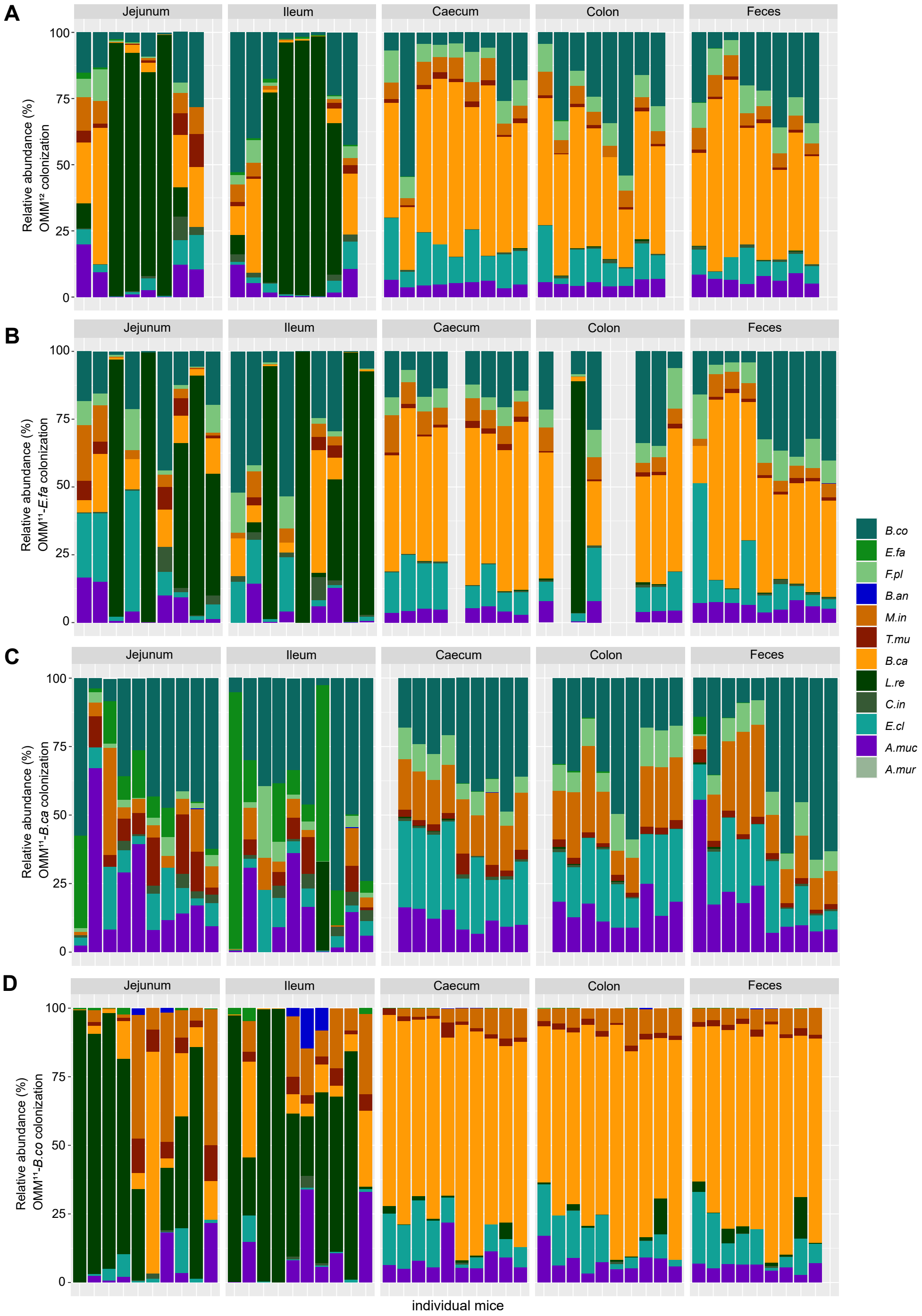

Fig. S12. Relative abundance of communities across the different regions of the murine gut. Germ-free C57B1/6J mice were inoculated with the full consortium (**A**) and the three dropout communities (OMM^{11}-*E. faecalis* (**B**), OMM^{11}- *B. caecimuris* (**C**), OMM^{11}-*B. coccoides* (**D**)). Absolute abundances of all strains 20 days after initial inoculation were determined as normalized 16S rRNA copies per g gut content. Based on absolute abdundances of strains in the different regions of the murine gut (jejunum, ileum, cecum, colon and feces), the relative abundances profiles were determined for the full consortium and dropout communities in each region. N=8-10 mice per group.

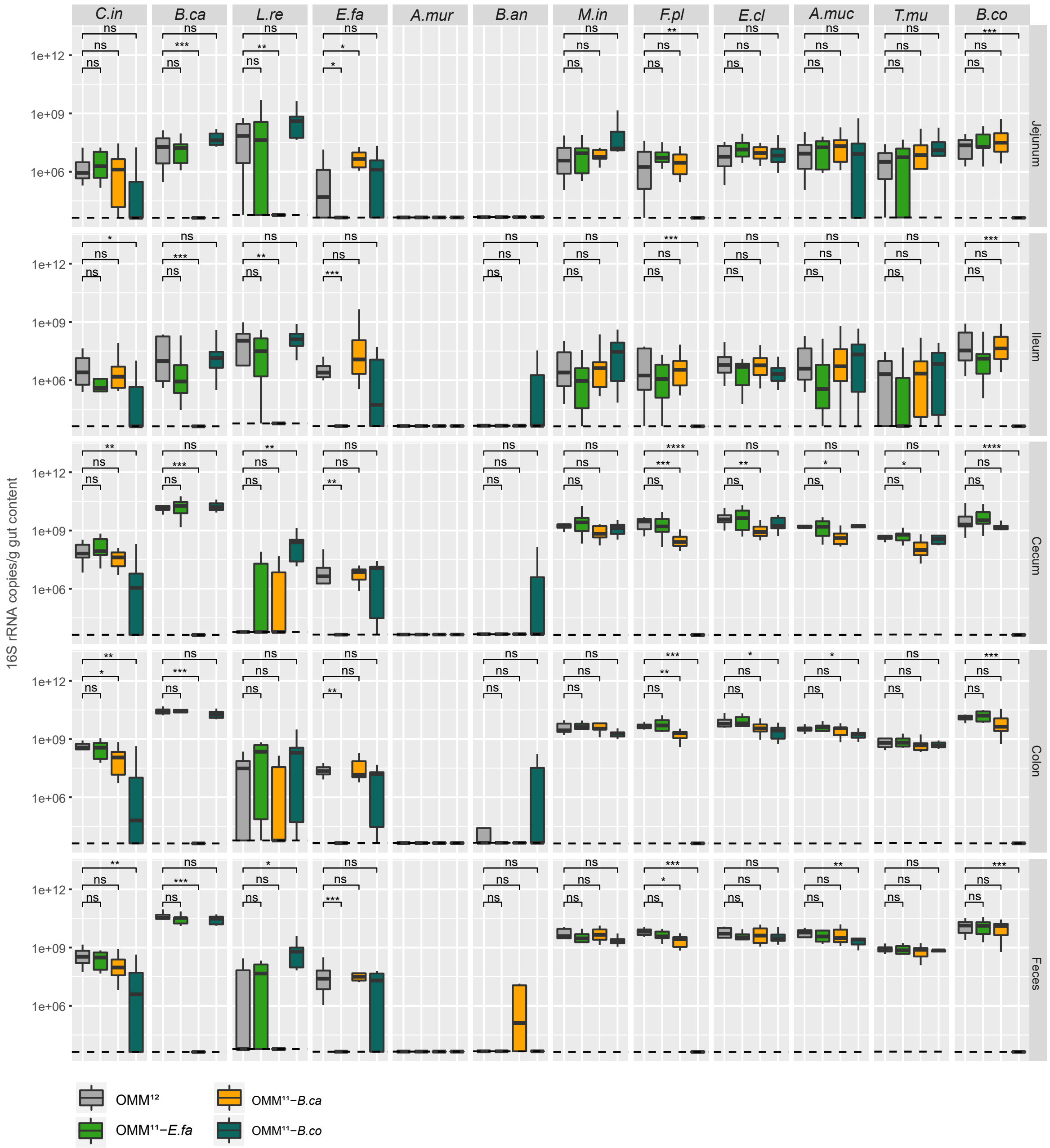

Fig. S13. Community assembly across the different region of the murine gut. Germ-free C57B1/6J mice were inoculated with the full consortium and the three dropout communities (OMM^{11}-*E. faecalis*, OMM^{11}-*B. caecimuris*, OMM^{11}-*B. coccoides*). Absolute abundances of all strains 20 days after initial inoculation were determined as normalized 16S rRNA copies per g gut content for each mouse in the different regions of the murine gut (jejunum, ileum, cecum, colon and feces, N=8-10 each). Median absolute abundances (black line) are shown with the corresponding upper and lower percentile (box, whiskers indicate 1.5 times interquartile range) for all individual strains. The strain specific detection limit is shown as dotted line. Using a two-sided Wilcoxon test, absolute abundances of the individual strains were compared between the full consortium and the corresponding dropout communities, p values are denoted as ns = not significant, $* < 0.05$, $** < 0.01$, $*** < 0.005$.

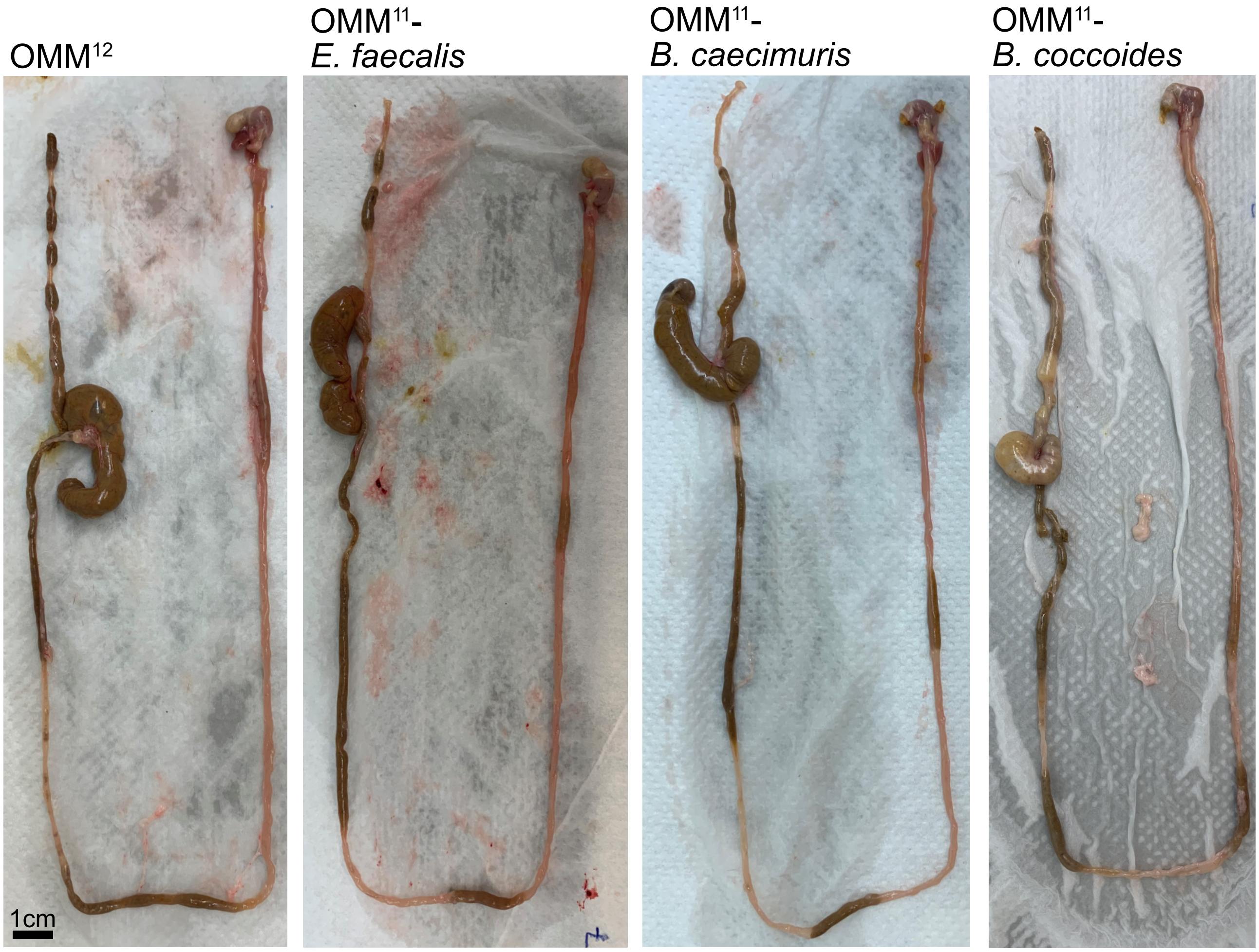

Fig. S14. Physiology of the GI tract of differently colonized mice. Exemplary sections of the gastrointestinal tract of individual mice for each group, differing in their bacterial colonization (OMM^{12}, OMM^{11}-*E. faecalis*, OMM^{11}- *B. caecimuris*, OMM^{11}-*B. coccoides*). Strongest difference are visible in cecum size and texture.

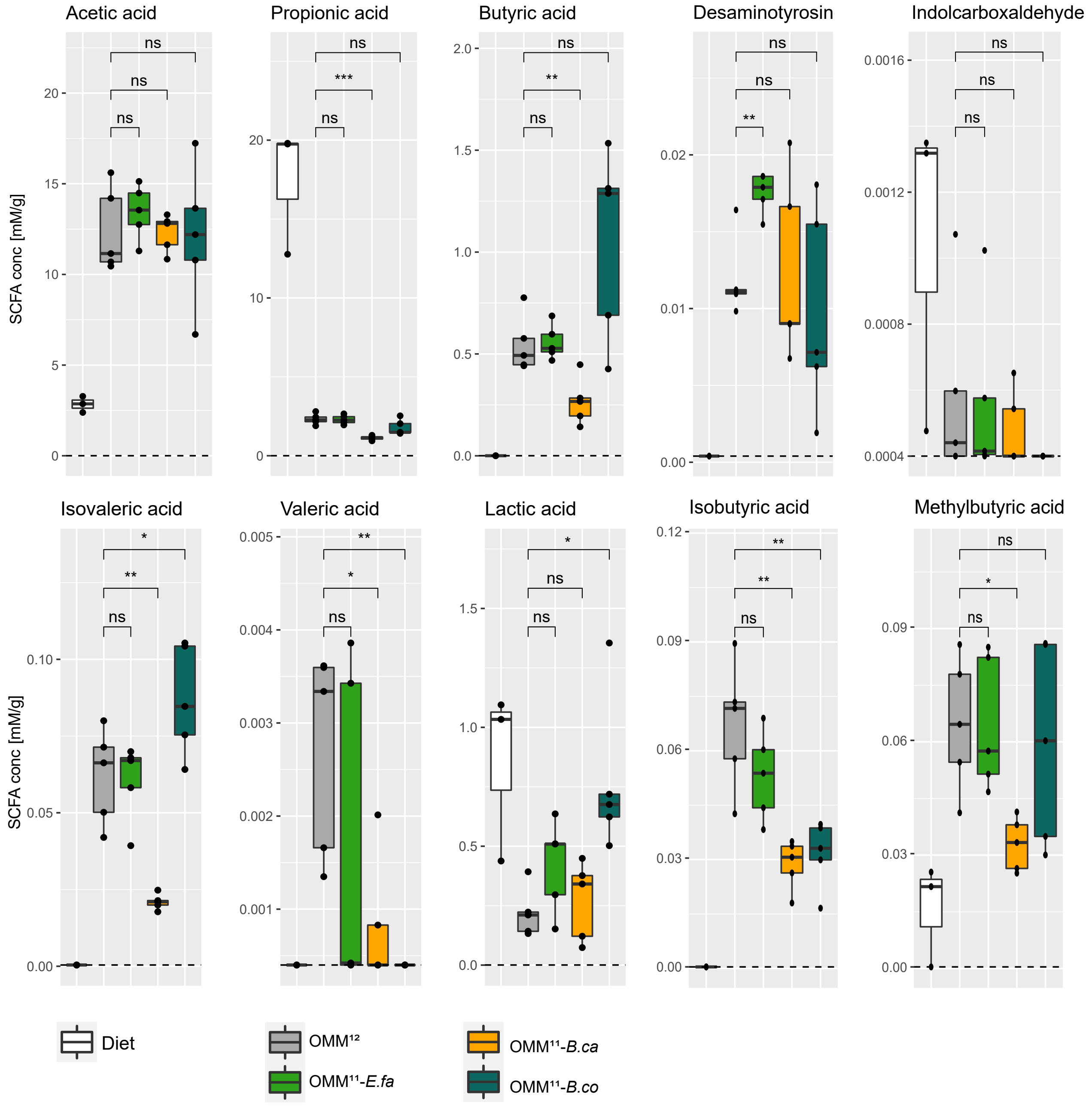

Fig. S15. SCFA concentrations in the murine cecum. SCFA concentrations were determined by targeted metabolomics analysis of cecal content of the differently colonized mice (N=5 per group) and are shown as median (black line) with the corresponding upper and lower percentile (box, whiskers indicate 1.5 times interquartile range). Using a two-sided t-test the SCFA concentrations in the cecum of mice colonized with the full consortium were compared to the cecum content of mice colonized with the individual dropout communities (OMM^{11}-*E. faecalis*, OMM^{11}- *B. caecimuris*, OMM^{11}-*B. coccoides*), p values are denoted as ns = not significant, * $<$ 0.05, ** $<$ 0.01.

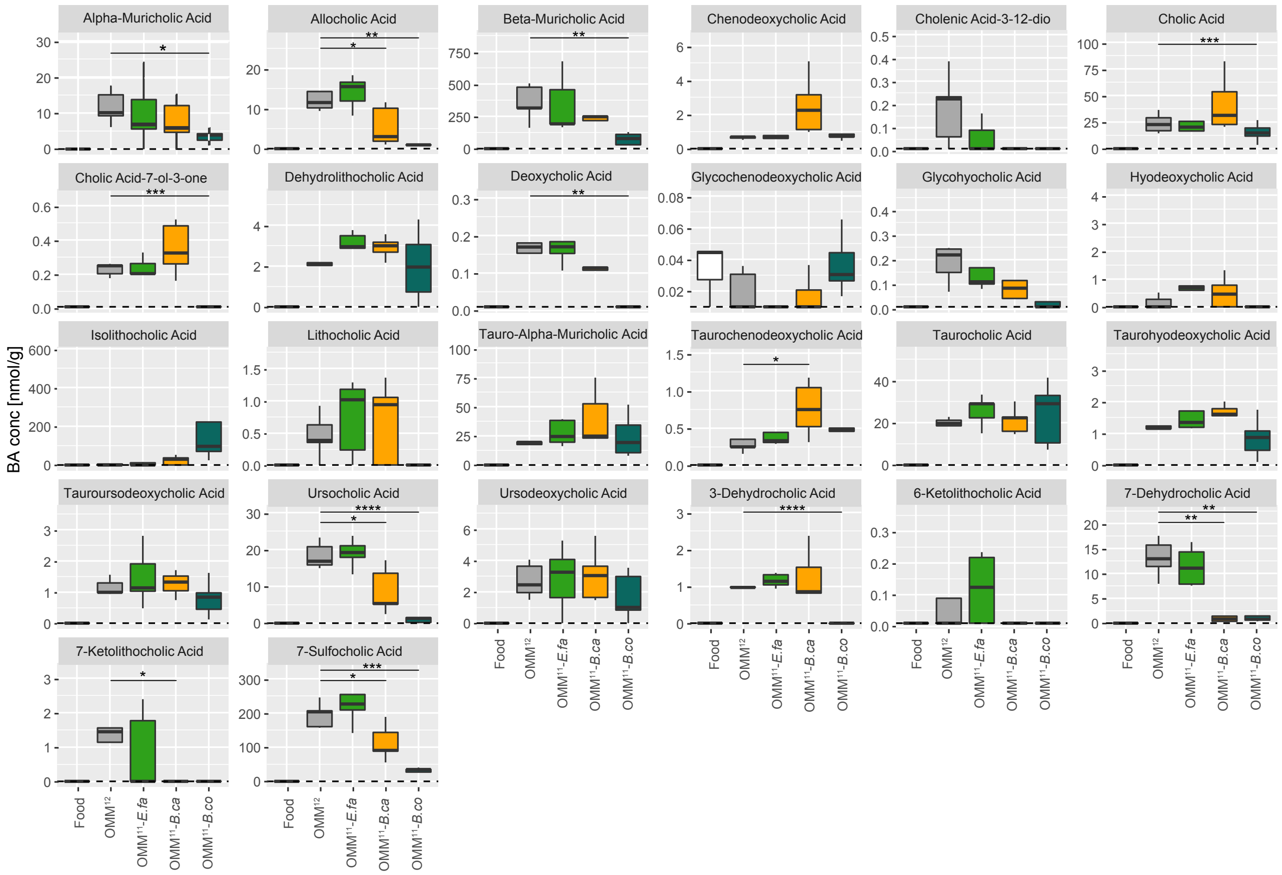

Fig. S16. Bile acid concentrations in feces from mice colonized with the full consortium and three dropout communities. Bile acid concentrations were determined by targeted metabolomics analysis of feces of the differently colonized mice (N=5 per group) and are shown as median (black line) with the corresponding upper and lower percentile (box, whiskers indicate 1.5 times interquartile range). Using a two-sided t-test the bile acid concentrations in the feces of mice colonized with the full consortium were compared to the feces of mice colonized with the individual dropout communities (OMM^{11}-*E. faecalis*, OMM^{11}- *B. caecimuris*, OMM^{11}-*B. coccoides*). p values are denoted as ns = not significant, * < 0.05, ** < 0.01, *** < 0.005, **** < 0.0001.

Supplemental Tables

	AF		APF		**GAM mod.** (Himedia)	TYG		YCFA	
component	*amount per l*	*company*	*amount per l*	*company*	*amount per l*	*amount per l*	*company*	*amount per l*	*company*
brain-heart infusion	18,5 g	Oxoid	18,5 g*	US Biological	-	-	-	-	-
trypticase soy broth	15 g	Oxoid	15 g*	US Biological	-	-	-	-	-
yeast extract	5 g	Roth	5 g	Roth	2.5 g	5 g	Roth	2.5 g	Roth
peptone	-	-	-	-	5 g	-	-	-	-
soya peptone	-	-	-	-	3 g	-	-	-	-
proteose petone	-	-	-	-	5 g	-	-	-	-
tryptone/pepton from casein	-	-	-	-	-	10 g	Roth	-	-
casitone	-	-	-	-	-	-	-	10 g	BD
meat extract	-	-	-	-	2.2 g	-	-	-	-
liver extract	-	-	-	-	1.2 g	-	-	-	-
digested serum	-	-	-	-	10 g	-	-	-	-
heat-inactivated fetal calf serum	3% (v/v)	Sigma-Aldrich	3% (v/v)	Sigma-Aldrich	-	-	-	-	-
D-glucose	0.5 g	Roth	-		0.5 g	2 g	Roth	2 g	Roth
1:1 mixture of arabinose[a], fucose[b], lyxose[b], rhamnose[b], xylose[c]	-	-	2.5 g	[a]Sigma-Aldrich, [b]TCI, [c]Roth	-	-	-	-	-
inulin	-	-	2 g#	Sigma-Aldrich	-	-	-	-	-
xylan	-	-	2 g#	Roth	-	-	-	-	-
starch	-	-	-	-	5 g	-	-	2 g	Roth
cellobiose	-	-	-	-	-	-	-	2 g	Roth
mucin	-	-	0.025%	Sigma-Aldrich	-	-	-	-	-
HCl-cysteine	0.5 g	Sigma-Aldrich	0.5 g	Sigma-Aldrich	0.3 g	0.5 g	Sigma-Aldrich	1 g	Sigma-Aldrich
L-arginine	-	-	-	-	1 g	-	-	-	-
L-trypthophan	-	-	-	-	0.2 g	-	-	-	-
hemin	1 mg	Sigma-Aldrich	1 mg	Sigma-Aldrich	0.01 g	-	-	10 mg	Sigma-Aldrich
menadione	0.5 mg	Sigma-Aldrich	0.5 mg	Sigma-Aldrich	-	1 mg	Sigma-Aldrich	-	-
vitamin K1	-	-	-	-	5 mg	-	-	-	-
hematin-histidine**	-	-	-	-	-	1 ml	Sigma-Aldrich	-	-
trace element/vitamins***	-	-	-	-	-	-	-	***	***
K_2HPO_4	2.5 g	Roth	2.5 g	Roth	-	100 ml 1M K_xPO_4	Roth	0.45 g	Roth
KH_2PO_4	-	-	-	-	2.5 g	-	-	0.45 g	Roth
$MgSO_4$-$7H_2O$	-	-	-	-	-	-	-	0.09 g	Sigma-Aldrich
Na_2CO_3	0.4 g	Merck	0.4 g	Merck	-	-	-	-	-
$NaHCO_3$	-	-	-	-	-			4 g	Sigma-Aldrich
NaCl	-	-	-	-	3 g	-	-	0.9 g	Roth
sodium thioglycollate	-	-	-	-	0.3 g	-	-	-	-
TYG salt solution****	-	-	-	-	-	40 ml	-	-	-
$CaCl_2$	-	-	-	-	-	8 mg	Sigma-Aldrich	0.09 g	Sigma-Aldrich
$FeSO_4$	-	-	-	-	-	0.4 mg	Sigma-Aldrich	-	-
resazurin	-	-	-	-	-	-	Sigma-Aldrich	1 mg	Sigma-Aldrich

* glucose-free

** 12 mg hematin dissolved in 10 ml 0.2M histidine solution (pH 8)

*** biotin (0.01 mg/l, Sigma-Aldrich), cobalamin (0.01 mg/l, Sigma-Aldrich), folic acid (0.05 mg/l, Sigma-Aldrich), p-aminobenzoic acid (0.03 mg/l, Sigma-Aldrich), pyridoxamine (0.15 mg/l, Fluka), thiamine (0.05 mg/l, Roth), riboflavin (0.05 mg/l, Sigma-Aldrich)

**** 0.05 g MgSO4-7H2O, 1 g NaHCO3, 0.2 g NaCl in 100 ml H2O

modified APF media: APF[inulin]: just with inulin, leave out xylan; APF[xylan]: just with xylan, leave out inulin; APF[mod]: without inulin and xylan

Table S1. Overview of media compositions. Comparison of composition of culture media used in this study. AF was used as described previously (Weiss et al, ISME J, 2022). APF medium was adapted from the recipe described previously (Weiss et al, ISME J, 2022) by replacing meat extract and supplements with glucose free BHI supplied by US Biological. Modified GAM medium (mGAM) is commercially available at Himedia (Himedia Labs). TYG and YCFA media were previously described (Whitacker et al., Cell, 2017 and Duncan et al., J Syst Evol Microbiol., 2002)

Potential polysaccharide degradation enyzmes for inulin and xylan in *B. caecimuris* I48

	Locus Tag	Genome position	Annotation on NCBI	PUL database ID	Word search
Inulinase	I5Q79_16515*	4035767-4037599	DUF4980 domain-containing protein	GH32 in PUL22: 2,6-beta-D-fructofuranosidase	
Xylanase	I5Q79_08890*	2096214-2098463	endo-1,4-beta-xylanase	-	endo-1,4-beta-xylanase
	I5Q79_10285	2505152-2509216	acetylxylan esterase	CE6 in PUL2: acetyl xylan esterase	-
	I5Q79_11165	2741693-2742679	glycoside hydrolase family 43 protein	GH43_31 in PUL40: beta-xylosidase	-
	I5Q79_11420	2831509-2833095	glycoside hydrolase family 30 protein	GH30_4/unk in PUL37: 1,4-beta-xylanase	-
	I5Q79_11750	2932024-2933343	glycoside hydrolase family 43 protein	GH43_31 in PUL35: 1,4-beta-xylanase	-
	I5Q79_16930	4133515-4134651	endo-1,4-beta-xylanase	-	endo-1,4-beta-xylanase
	* = Fig. 4A				

Sources	Link	Last visit
B. caecimuris I48 genome	https://www.ncbi.nlm.nih.gov/nuccore/CP065319	4th October 2022
PUL database cazy.org	http://www.cazy.org/PULDB/index.php?sp_name=Bacteroides+caecimuris+I48&sp_ncbi=	4th October 2022

Table S 2. Screen for polysaccharide degradation enzymes in *B.caecimuris*. The genome of *B.caecimuris* was screened for polysaccharide utilization loci (PUL) specific for inulin and xylan degradation found in literature (Chijiwa et al., Microbiome, 2020; Despres et al., BMC Genomics, 2016; Lapebie et al., NatCom, 2019) and a PUL database (Methods). Sequences of key enzymes for inulin and xylan degradation were blasted against the *B. caecimuris* genome and names of key enzymes were checked in genome annotations of *B. caecimuris* via word search ("1,4-beta-xylanase", "beta-xylosidase", "inulinase" and similar versions). Identified locus tags, annotations and PUL database IDs are listed.

PERMANOVA of Bray-Curtis dissimilarities
adjusted p-value (Benjamini-Hochberg)

	OMM^{11}-*E.fa* vs. OMM^{12}	OMM^{11}-*B.ca* vs. OMM^{12}	OMM^{11}-*B.co* vs. OMM^{12}
Jejunum	0.6895	0.0099	0.4518
Ileum	0.4693	0.0648	0.4693
Cecum	0.512	0.0003	0.072
Colon	0.4502	0.00015	0.00015
Feces	0.9906	0.0003	0.0021

Table S3. Statistical analysis of Bray-Curtis dissimilarities based on absolute strain abundances in the murine gut. Bray-Curtis dissimilarities analysis of absolute strain abundances in the different regions of the murine gut was performed on samples obtained from mice colonized with the full consortium and communities lacking the three identified keystone species *E. faecalis*, *B. caecimuris*, *B. coccoides* for the individual mice (N=8-10 mice per group). For each region, pairwise comparison of Bray-Curtis dissimilarities of the individual dropout consortia to the full consortium was performed using a two-sided permutational multivariate analyses of variance (PERMANOVA) in R using the function Adonis (method "bray" with 9,999 permutations). Obtained p values were adjusted using the Benjamini-Hochberg method. Significant values ($p < 0.05$) are highlighted in red.

Primer name	Sequence	Amplification target
P149	gcaaattggtatttcacagtcc	L50A-L50B
P150	catctaacaacctcttcttttattctt	
P151	caaagacaacacgggataacactc	O16
P152	gtcgttaactttcacaaaatgtaagtc	
P153	cgaatttttagtttcggctcttt	Ent96
P154	cgtttcaattaatgacctagacttc	
P159	ggaaaacttcacgtatcggatc	Arm 1 L50A-L50B
P160	ggtggtgtcgacaatatatctcctccaattattttttgttc	
P161	ggtggtgtcgacttttatgatatattaaatttttaaagagactatga	Arm 2 L50A-L50B
P162	ggtggtctgcaggtttgaaccagacctgcaat	
P163	ggtggtggatccgatgattggtgggttagtagtagg	Arm 1 Ent96
P164	ggtggtgtcgacagtattatctcctttcgtcctctcc	
P165	ggtggtgtcgacaaatttctaattagaataaccgtcctc	Arm 2 Ent96
P166	ggtggtctgcagtccgtaagggcgaattattta	
P167	ggtggtggatccctcttggcttatcaaatttggaa	Arm 1 O16
P168	ggtggtgtcgacaaataaatccctactttctttttcctt	
P169	ggtggtgtcgactgaatttaaaagatcatgttacgga	Arm 1 O16
P170	ggtggtctgcagcggtatatccttgctgagctttt	
pLT06_FW_b	cgtgtgctctacgaccaaaact	pLT06 vector
pLT06_RV_b	tcctcctttctattttgattag	

Table S4. List of primers for the construction of exchange vectors for the engineering of *E. faecalis*. Vector pLT06 was used for the deletion of enterocins L50A-L50B, Ent96 and O16 in *E. faecalis* strain. DNA fragments of 500-1000 bp upstream and downstream of the gene targeted for deletion (homologous arms 1 and 2) were amplified by PCR, using primers to insert restriction sites. BamHI and SalI restriction sites were added to arms 1, and SalI and PstI sites were added to arms 2. The PCR products of the arms were digested using the appropriate restriction enzymes and ligated with pLT06.
